# Semiparametric change points detection using single index spatial random effects model in environmental epidemiology study

**DOI:** 10.1371/journal.pone.0315413

**Published:** 2024-12-12

**Authors:** Hamdy F. F. Mahmoud, Inyoung Kim

**Affiliations:** 1 Department of Statistics, Virginia Polytechnic Institute and State University (Virginia Tech), Blacksburg, VA, United States of America; 2 Department of Statistics, Mathematics, and Insurance, Assiut University, Asyut, Egypt; RMIT University, AUSTRALIA

## Abstract

Environmental health studies are of great interest in research to evaluate the mortality-temperature relationship by adjusting spatially correlated random effects as well as identifying significant change points in temperature. However, this relationship is often not expressed using parametric models, which makes identifying change points an even more challenging problem. This paper proposes a unified semiparametric approach to simultaneously identify the nonlinear mortality-temperature relationship and detect spatially-dependent change points. A unified method is proposed for the model estimation, spatially dependent change points detection, and testing whether they are significant simultaneously by a permutation-based test. We operate under the assumption that change points remain constant, yet acknowledge the uncertainty regarding their precise number. These change points are influenced by the smoothing of an unknown function, which in turn relies on a smoothing variable and spatial random effects. Consequently, the detection of change points may be influenced by spatial effects. In this paper, several simulation studies are conducted to evaluate the performance of our proposed approach. The advantages of this unified approach are demonstrated using epidemiological data on mortality and temperature.

## Introduction

For centuries, the effects of weather and global warming on people have been a public health concern. Previous studies [1–3] have indicated that the temperature-mortality relationship can be depicted as a U, J, or V curve; that is, episodes of extremely hot or cold temperatures increase mortality. The lowest end of the curve was defined as the minimum mortality temperature or the change point—that is, the temperature of the lowest mortality. Extreme temperatures increase the heart rate because of the increase of blood flow from the body to the skin, which can lead to shaking in cold temperatures or sweating in high temperatures. The human body has multiple thermoregulatory mechanisms to counter extreme heat and cold conditions to keep temperature homeostasis within normal values. When temperature change occurs within certain ranges, the human body can adapt and allow individuals to follow some physical and mental activities, but exposure to temperature extremes outside these ranges for a long period is a risk to human health and can result in mortality. According to [4], elevated mortality rates correlate with high temperatures, primarily attributed to illnesses such as cerebrovascular, cardiovascular, and respiratory diseases. This phenomenon is attributed to the effect of hot temperatures on raising blood cholesterol and viscosity levels.

Climate change is a serious public health issue, and specific policies to reduce the effects of heat waves would be appropriate for public policy. These policies need to target successful interventions and populations that are vulnerable. One of the possible mitigation strategies for this is using air conditioning. Because climate change will likely increase the mean temperature, as well as the frequency of heat events, it is very important to evaluate the links between human health and climate, to better identify populations at risk and take preventive measures. As mean temperatures continue to rise in the future, the issue of heat-related mortality is poised to escalate. By delving into the connection between temperature and mortality rates, as well as identifying change points within cities, we can enhance awareness surrounding hot weather as a significant environmental hazard.

### One city

Many articles have studied the mortality-temperature relationship in a specific area or city [5–10]. In these studies, the nonlinear mortality function is first estimated by the generalized linear model and then the change point is detected by observing the temperature degree that is associated with the minimum risk. No testing of whether the change point is statistically significant is considered. [11] studied the mortality-temperature function in a single city, Seoul City, South Korea, using the single index model and tested the significance of the change point by a permutation-based test.

### Multiple cities

Some articles [12, 13] have studied multiple cities and have found that change points were associated with temperature and they varied by location, especially with latitude, people who live in cities at higher latitudes have lower thresholds for ambient temperature, whereas people who live in cities at lower latitudes have higher thresholds for ambient temperature [4, 14–16]. In these studies, the generalized additive model is used to estimate the temperature-mortality relationship for each city separately, and the minimum mortality risk or AIC criterion is used to find the change point. Other studies have used a distributed lag nonlinear model to estimate the relationship in each city. [17–20] studied 15 European cities, 63 cities in five East-Asian Countries, 47 Japanese cities, and 31 Chinese cities, respectively. After estimating the relationship in each city separately, the change point is estimated as the temperature that is associated with minimum mortality or maximum likelihood.

However, these studies have not fully addressed (1) whether the change points are accurately detected and tested in multiple cities cases, (2) whether spatial random effect plays an important role in the model, and (3) whether the model assumption is flexible in terms of the link function compared to the single index model when multiple cities considered.

### Problem and objectives

This paper introduces a unified semiparametric approach to simultaneously identify the nonlinear mortality-temperature relationship, and detect and test spatially-dependent change points. This approach includes a proposed model and a permutation test. To the best of our knowledge, no such model has been introduced in the statistical literature. We refer to this model as the “semiparametric change points single index spatial random effects model” (CP-SISM). The proposed approach has the following four characteristics:

1. Spatial random effects are incorporated into the model not only because ignoring random effects may mask the true form of the mortality-temperature relationship due to aggregating the data of all cities, but also to make the proposed model able to predict mortality at new locations. The six cities in our motivating data are located close to each other due to the size of South Korea, so we assume that the correlation between spatial effects exists. Hence, the detection of change points can be affected by spatial effects. Previous work studied each city separately without including the spatial effect.
2. The model is flexible in terms of the link between the response variable and the mean function. A semiparametric approach, based on the single index model, is employed to simultaneously estimate the nonlinear mortality-temperature relationship while adjusting for weather variables. The single index model is chosen because it combines parametric and nonparametric components, offering a flexible representation of real data and enabling the proposed model to effectively describe nonlinear relationships. This approach also helps avoid misleading results that can arise from selecting an incorrect link function. Previous studies often utilized generalized linear models or additive models to estimate the temperature-mortality relationship.
3. The change points are included in the nonparametric function to ensure accurate detection. In the proposed model, change-point parameters are incorporated into the single index function because smoothing the unknown mean function may impact change-point detection. In previous studies, the change point was typically selected based on certain criteria after estimating the temperature-mortality relationship. However, the change point detected using this method is influenced by the smoothing of the function.
4. The permutation-based change-points detection procedure is introduced to test the significance of the detected change points under the CP-SISM. The previous work smoothed the nonparametric function and selected the change point that has minimum mortality or is based on some criteria, such as AIC or BIC. The permutation test is more powerful and robust compared to other tests/criteria-based analyses.

The remainder of this paper is organized as follows. In Section, the motivating data of this study is introduced. In Section, the proposed model is presented. A simultaneous procedure for estimating the proposed model while detecting and testing the significance of spatially dependent change points based on a permutation test is introduced in Section. In Section, several simulation studies are conducted. Section considers applying our unified method to South Korea’s real data. Section includes discussion and conclusion.

## Data and motivation

In our motivating data, non-accident mortality and weather variables, such as mean pressure, mean temperature, mean humidity, and time, were recorded daily from January 2000 to December 2007 for six major cities in South Korea (Seoul, Busan, Daegu, Incheon, Gwangju, and Daejeon). In total, the data comprise 2922 observations for each city. In addition, weekly data are obtained where daily weather variables were averaged such that each city has 417 observations. Because those cities are different in population size, the weekly non-accident mortality of each city is divided by the population size and multiplied by 1 million to obtain weekly nonaccident mortality per 1 million persons for each city. The numerical summary statistics of the weather variables of each city are presented in S1 Table of the supporting information.

**Fig 1:**
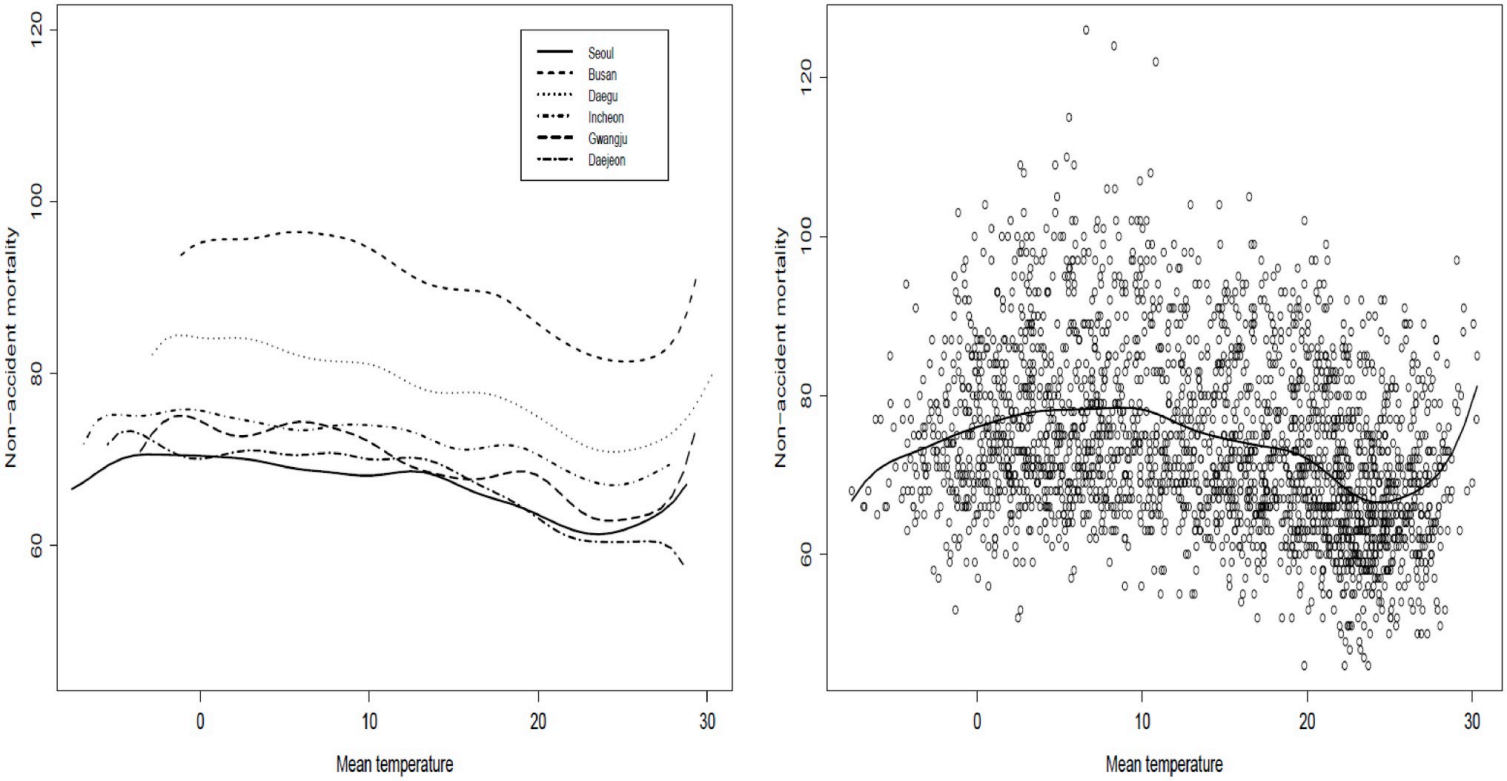
The aggregated smoothed mortality-temperature function of all cities along with scatter plot (a), and smoothed mortality-temperature functions of the six major areas in Korea (b).

**Fig 2:**
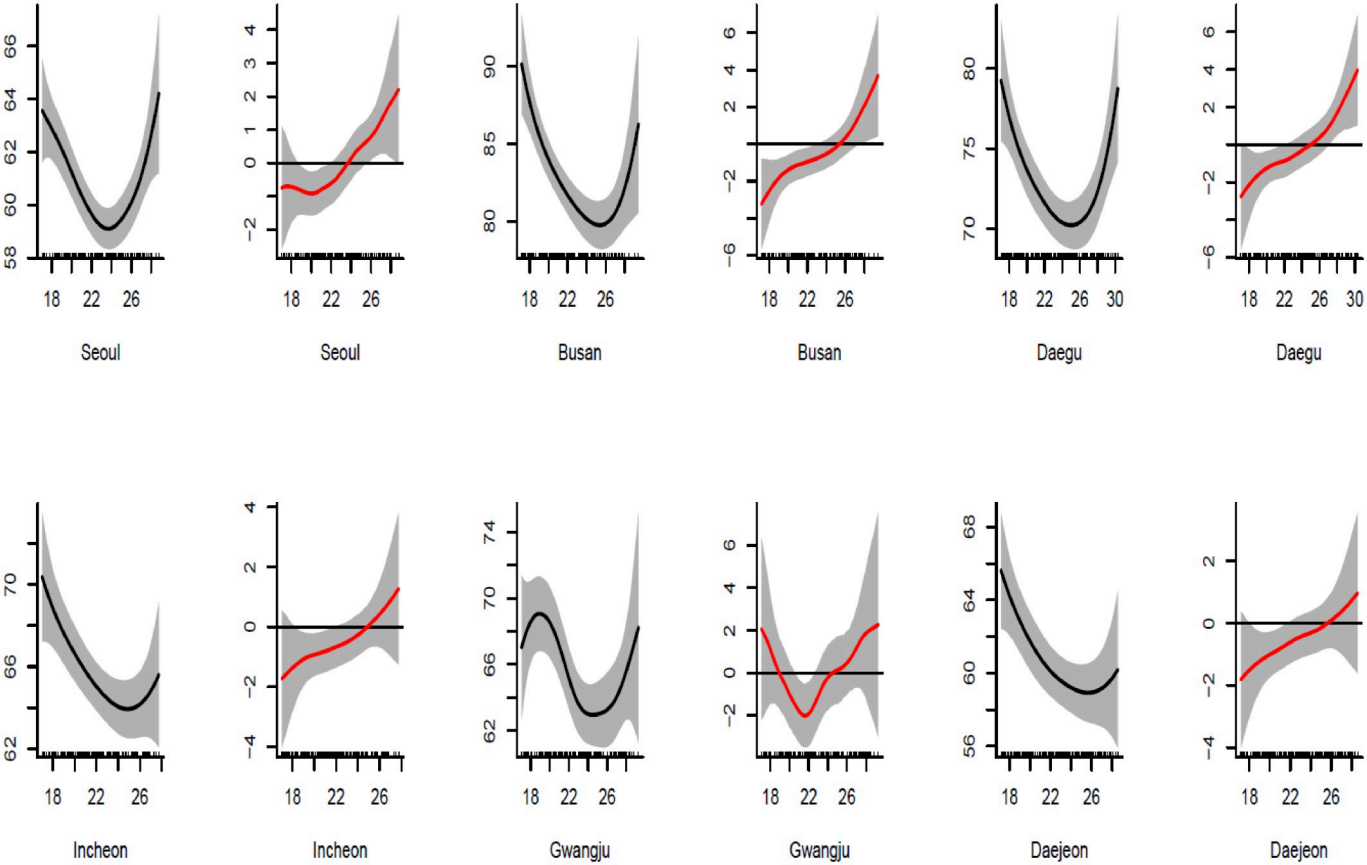
Spline smoothed mortality-temperature function of each city along with the smoothed derivative function. The black line represents the smoothed temperature-mortality function (the x-axis is the mean temperature and the y-axis is the smoothed mean temperature) and the red line is the derivative function of the temperature-mortality function (the x-axis is the mean temperature and the y-axis is the derivative of the mean temperature-mortality).

Fig 1(a) shows a common change point of aggregated data of the six cities. Fig 1(b) reveals that the smoothed functions of non-accident mortality and temperature are similar in shape and show change points of all cities compared to each other. It shows there are possible change points in four cities (Seoul, Busan, Daegu, and Gwangju) and for the other two cities, it is not clear. That is because detecting a change point is affected by smoothing the unknown function, and change points commonly close to the boundaries where the smoothed function is located are not accurate. By focusing only on the interval that has a possible change point, Fig 2 shows that each city has a possible change point at some degree of temperature. These change points need to be studied simultaneously to see whether they are spatially dependent after adjusting the relationship by the weather variables. One important question here is whether these change points are statistically significant and/or significantly different from each other (i.e., spatially dependent). Hence, we study two cases by introducing a semiparametric model: a common change point (change points are not spatially dependent) and different change points over locations (spatially dependent).

## Semiparametric change points single index spatial random effects model

Let *Y*_*is*_ be the *i*th observation at the *s*th city (location/region), and let *x*_*jis*_ be the *i*th value of the *j*th explanatory variable at city *s*, where *i* = 1, …, *n*, *s* = 1, …, *r*, and *j* = 1, …, *p*. Here, *n*, *r*, and *p* denote the total number of observations, the number of locations, and the number of explanatory variables, respectively. Let $\left(\theta_{s}^{1},\ldots ,\theta_{s}^{L}\right)$ denote the possible multiple *L* change points at city *s* and ${\left[x_{1is}-\theta_{s}^{l}\right]}_{+}=\text{max}\left(0,x_{1is}-\theta_{s}^{l}\right)$ and *l* = 1, …, *L*. We denote *f*(⋅) as the unknown mean function of the response variable. Let *u*_*s*_ be the spatial random effect associated with the *s*th city that follows a Gaussian process (GP) with covariance matrix *Ω*, and let *ϵ*_*is*_ be the random error associated with the *i*th observation at city *s*. We further denote a probability density/mass function of *y*_*s*_ as *p*_*d*_(*y*_*s*_|*μ*_*s*_, *u*_*s*_). The CP-SISM can be written as

$$\begin{array}{rrr} y_{is}|\mu_{s},u_{s} & \sim & p_{d}(y_{s}|\mu_{s},u_{s}),\\ \mu_{s}|u_{s} & = & f(\beta_{1}x_{1is}+\beta_{11}{\left[x_{1is}-\theta_{s}^{1}\right]}_{+}+\ldots +\beta_{1L}{\left[x_{1is}-\theta_{s}^{L}\right]}_{+}\\ \; & \; & +\beta_{2}x_{2is}+\beta_{3}x_{3is}+\ldots +\beta_{p}x_{pis})+u_{s},\\ u_{s} & \sim & GP(0,\sigma_{u}^{2}\Omega ), \end{array}$$ (1)

where

- the spatial effect, *u*_*s*_ (*s* ∈ *R*^2^), follows a Gaussian stationary process with mean **0** for all *s* and a variance-covariance matrix depends only on the distance between any two locations *s* and *s* + *a*; $\text{cov}\left(u_{s},u_{s+a}\right)=C\left(a\right)$ for all *s*, *a* ∈ *R*^2^, where *C*(⋅) is a parametric covariance function, and *a* is the distance between two cities;
- ***β*** = (*β*_1_, *β*_11_, …, *β*_*p*_) represents the vector of the single index coefficient parameter and $\theta_{s}=\left(\theta_{s}^{1},\ldots ,\theta_{s}^{L}\right)$ denotes the unknown parameters for multiple change points at city *s*;
- Given *u*_*s*_ and ***θ***_*s*_, $y_{s}$ follows the Poisson distribution (Pois) with mean $E(y_{s}|u_{s},\theta_{s})$.

In matrix form, this model (1) can be written as

$$\begin{array}{ccc} y|\mu ,u & ∼ & \text{Pois}(\mu |u,\alpha ),\\ \mu |u,\alpha & = & f(X(\theta )\beta )+Zu, \end{array}$$ (2)

where $X=\left[x_{1},{\left(x_{1}-Z\theta \right)}_{+},x_{2},x_{3},\ldots ,x_{p}\right]\equiv X\left(\theta \right)$ is a *rn* × (*p* + *L*) matrix of regressors’ values, ***α*** = (***θ***, ***β***)^*T*^ is a (*p* + *L*) × 1 vector of parameters, Z is a *nr* × *r* matrix of 1s, **u** is a vector of unobservable spatial correlated random effects, $u∼\text{MN}(0,\sigma_{u}^{2}\Omega )$, where $\sigma_{u}^{2}$ is the variance of spatial effects and *Ω* is a known parametric covariance function that depends on the distance between two cities. The random process is assumed to be stationary and isotropic, and the covariance between two cities depends on the distance between them.

Spatial Gaussian Processes provide a robust, flexible, and interpretable approach for spatial modeling, especially when dealing with continuous spatial variation and complex dependencies. Their adaptability, particularly in terms of covariance functions and Bayesian compatibility, makes them a superior choice in many contexts compared to SAR, CAR, or traditional kriging methods, which may impose more restrictive assumptions on the spatial data, [21–24].

More specifically, for our motivating real data, which has non-accident mortality as the response variable, we can write CP-SISM as the following:

$$\begin{array}{lll} y_{s}|\mu_{s} & \sim & \text{Pois}(\mu_{s}|u_{s},\beta ,\theta_{s});\\ \mu_{s}|u_{s},\beta ,\theta_{s} & = & f(\beta_{1}x_{1s}+\beta_{11}{\left[x_{1s}-\theta_{s}^{1}\right]}_{+}+\ldots +\beta_{1L}{\left[x_{1is}-\theta_{s}^{L}\right]}_{+}\\ \; & \; & +\beta_{2}x_{2s}+\beta_{3}x_{3s}+\ldots +\beta_{p}x_{ps})+u_{s}. \end{array}$$ (3)

The unknown function, *f*(⋅), spatial effect, *u*_*s*_, single index coefficients parameters, ***β***, and the vector of change points, ***θ***, need to be estimated simultaneously and to be tested as to whether the change points are significant. The model parameters estimation needs a restriction on the single index coefficient parameters to fix the identifiability problem. A possible restriction is to set one of the parameters of ***β*** to be equal to 1 [25, 26] or to use ‖***β***‖ = 1 [27–29].

This restriction prevents parameters from taking values that lead to indistinguishable outcomes, enabling the model to have a unique solution and thus be identifiable. Additionally, it reduces the model’s complexity, preventing it from overfitting to noise in the data. This is particularly important in high-dimensional settings, as it stabilizes the estimation process by narrowing the parameter space. It also produces a simpler model, making it easier to interpret the impact of each coefficient, and helps the optimization algorithm converge more quickly and accurately, avoiding issues like local minima or divergence during estimation.

This model has several advantages: (1) It enables us to incorporate spatial effects into the model, (2) it enables us to detect multiple change points for each city, (3) it avoids the curse of the dimensionality problem by using the single index function, and (4) it is more flexible compared to the parametric models.

## Change-point detection and testing

In this section, we propose a testing procedure to identify the significant spatially dependent change points. This procedure consists of an estimation step and a test step. These two steps are iterated until significant change points are detected if they exist. The estimation step for CP-SISM is based on an adjusted Monte Carlo Expectation Maximization (MCEM) algorithm. The EM algorithm consists of two steps: expectation (E-step) in which the spatial effects are estimated ($u=u_{1},u_{2},\ldots ,u_{r}$) and maximization (M-step) in which the variance of the spatial effects ($\sigma_{u}^{2}$) is estimated. The vector of the coefficient parameters (***β*** = *β*_1_, …, *β*_*p*_) is estimated using the Ichimora method, and the *f*(*index*) function is estimated using a smoothing method, such as the kernel method.

The estimation of *f*(⋅) using the Ichimura method is performed as follows:

**Step 0**: For a given estimate of the index coefficient vector ***β***, we compute the single-index values *Z*_*i*_ = *X*_*i*_***β***, where *i* = 1, …, *n*.
**Step 1**: The unknown function *f*(⋅) is estimated using *kernel smoothing*. Specifically, for any value *z*, *f*(*z*) is estimated as:
  $$\begin{array}{c} \hat{f}\left(z\right)=\frac{\sum_{i=1}^{n}K_{h}\left(z-Z_{i}\right)y_{i}}{\sum_{i=1}^{n}K_{h}\left(z-Z_{i}\right)}, \end{array}$$
  where:*K*_*h*_(⋅) is a kernel function with a bandwidth *h*, *y*_*i*_ are the observed responses, and *Z*_*i*_ = ***β***^⊤^*X*_*i*_ are the single-index values. This approach smooths the observed *y* values as a function of the single-index *Z*, providing an estimate of *f*(⋅).
**Step 2**: The estimation of *f*(⋅) and ***β*** is performed iteratively. After updating ***β*** using optimization techniques, *f*(⋅) is re-estimated based on the updated single-index values until convergence is achieved.

To estimate a change point, a grid search is used. At each possible change point, the EM algorithm is run to estimate the spatial effects and model parameters, and the test procedure is used to see whether it is a significant change point. So the order of the estimation is as follows: at each possible change point, the EM algorithm is used to estimate the spatial effects and variance of spatial effects, and then model parameters and the unknown function are estimated using the Ichimura method. For each possible change point, the sum of the squared residuals is calculated and the change point associated with the minimum sum of squared residuals is selected and is then tested to determine whether it is significant based on the calculated p-value of the permutation test.

### Estimation step

The estimation step of CP-SISM is based on an adjusted MCEM algorithm. The EM algorithm consists of an expectation (E-step) and a maximization (M-step). Incorporating the Monte Carlo step into the EM algorithm gives the MCEM algorithm, which is commonly used in the generalized linear mixed models estimation [30–35].

Our proposed model, CP-SISM, has the following complete-data log-likelihood form:

$$\begin{array}{c} \text{log}f_{y_{s},u_{s}}\left(y_{s},u_{s}|\mu_{s},\sigma_{u}^{2},\Omega \right)=\text{log}f_{y_{s}}\left(y_{s}|\mu_{s},u_{s}\right)+\text{log}f_{u_{s}}\left(u_{s}|\sigma_{u}^{2}\Omega \right), \end{array}$$ (4)

where $y_{s}∼\text{Pois}\left(\mu_{s}|u_{s}\right)$, $u_{s}∼GP\left(0,\sigma_{u}^{2}\Omega \right)$, ***μ***_*s*_|*u*_*s*_ = *f*(*X*_*s*_***β***) + *u*_*s*_, $\Omega =\text{Cov}\left(u_{s},u_{s+a}\right)={\text{exp}(||a||}^{2}/\rho_{u})$ for all *s*, *a* ∈ *R*^2^, *a* is the distance between two cities *s* and *s* + *a*, and *ρ*_*u*_ is the dependence range.

In the E-step of the MCEM algorithm for our model estimation, there is no closed form available. Hence, random samples are generated from the full conditional distribution of **u** using Bayesian MCMC. The single-component Metropolis-Hastings (M-H) algorithm is used, i.e., a single component is updated at each iteration, say the *s*th component, *u*_*s*_. Selecting a proposal function is essential in the M-H algorithm. Because the spatial random effects are correlated, we propose generating candidate values from the conditional normal distribution $N(\bar{\gamma },\sigma_{0}^{2}\bar{\Omega })$. The following illustrates the derivation of the conditional normal distribution. Let $v=\left(v_{1},v_{2},\ldots ,v_{n}\right){=(v_{1},v_{2})}^{T}$ have multivariate normal distribution with mean ***γ*** = (*γ*_1_ ***γ***_2_)^T^ and variance-covariance matrix $\sigma_{0}^{2}\Omega$, where

$$\begin{array}{c} \Omega =(\begin{array}{cc} \sigma_{11} & \Sigma_{12}\\ \Sigma_{21} & \Sigma_{22} \end{array}). \end{array}$$

The distribution of *v*_1_, given that $v_{2}=a$, is a multivariate normal distribution $\left(v_{1}|v_{2}=a\right)∼N\left(\bar{\gamma },\sigma_{0}^{2}\bar{\Sigma }\right)$, where $\bar{\gamma }=\gamma_{1}+\Sigma_{12}\Sigma_{22}^{-1}\left(a-\gamma_{2}\right)$ and variance-covariance matrix $\bar{\Sigma }=\sigma_{11}-\Sigma_{12}\Sigma_{22}^{-1}\Sigma_{21}$. So the conditional normal distribution, $N(\bar{\gamma },\sigma_{0}^{2}\bar{\Sigma })$, is our proposal distribution, where $\sigma_{0}^{2}$ is the proposal variance of the correlated spatial random effects.

Similarly, given the other spatial random effects, we obtain the conditional normal distribution of *u*_*s*_ as $N(\bar{\gamma },\sigma_{u}^{2}\bar{\Omega })$, where $\bar{\gamma }=\Omega_{12}\Omega_{22}^{-1}\left(a\right)$ and $\bar{\Omega }=\sigma_{11}-\Omega_{12}\Omega_{22}^{-1}\Omega_{21}$. As a result, the acceptance probability, in E-step, can be written as

$$\begin{array}{c} \text{min}[\frac{f\left(y_{s}|u_{s}^{*},\mu_{s}\right)f_{u}\left(u_{s}^{*}|\bar{\gamma },\sigma_{u}^{2}\bar{\Omega }\right)}{f\left(y_{s}|u_{s},\mu_{s}\right)f_{u}\left(u_{s}|\bar{\gamma },\sigma_{u}^{2}\bar{\Omega }\right)},1], \end{array}$$ (5)

where $f_{u_{s}}\left(u_{s}|\bar{\gamma },\sigma_{u}^{2}\bar{\Omega }\right)$ is the conditional distribution of *u*_*s*_ that is given all the other spatial random effects.

In M-step, given spatial effects and candidate spatial change points, $\sum \text{log}f(u|\sigma_{u}^{2}\Omega )$ is maximized to obtain ${\hat{\sigma }}_{u}^{2}$, estimate ***β***, $\hat{\beta }$, and smooth the function *f*(⋅) to obtain $\hat{f}\left(\cdot \right)$. Then E-step and M-step are iterated until the convergence is achieved.

### Testing step

In this section, we explain how to conduct the testing procedure by connecting a nonparametric Poisson regression with a single index nonparametric function *f*(⋅) that can estimate the link function as well. In Poisson regression with an unknown function *m*(⋅) and a link function *g*(⋅), we can express the model as

$$\begin{array}{lll} g\{E(y|\mu )\} & = & m\left\{X\left(\theta \right)\beta \right\}+Zu;\\ E(y|\mu ) & = & g^{-1}\left[m\left\{X\left(\theta \right)\beta \right\}+Zu\right];\\ \; & = & g^{-1}\left[m\left\{X\left(\theta \right)\beta \right\}\right]\times g^{-1}\left(Zu\right);\\ \; & = & f\left\{X\left(\theta \right)\beta \right\}\times g^{-1}\left(Zu\right);\\ \; & \approx & \left[f\left\{X\left(\theta \right)\beta \right\}+c\right]\times [g^{-1}\left(0\right)+{\left\{g^{-1}\left(0\right)\right\}}^{\prime }Zu+O\left(\left|\left|Zu\right|\right|\right)];\\ \; & = & f\left\{X\left(\theta \right)\beta \right\}g^{-1}\left(0\right)+c{\left\{g^{-1}\left(0\right)\right\}}^{\prime }Zu+O\left(\left|\left|Zu\right|\right|\right). \end{array}$$ (6)

Because *g*^−1^(0) and *c*{*g*^−1^(0)}′ are both constants, they can be merged to the unknown function *f*(⋅) and random variable *u*. Hence, we can develop the testing procedure under the following approximated model,

$$\begin{array}{c} y\approx f(X(\theta )\beta )+Zu+\epsilon \end{array}$$ (7)

where ***ϵ*** = **y** − ***μ***. Hence, our permutation testing procedure is developed under this approximation.

The multiple spatially dependent change-point candidates in cities, $\theta =(\theta_{s}^{1},\theta_{s}^{2},\ldots ,\theta_{s}^{L})$, *s* = 1, …, *r*, are tested to determine whether they are significant based on our permutation-based testing approach described as follows.

Under the null hypothesis of no change points, CP-SISM can be written as

$$\begin{array}{lll} y|\mu^{\left(0\right)} & \sim & \text{Pois}(\mu^{\left(0\right)}|\beta ,u),\\ \mu^{\left(0\right)} & = & f(\beta_{1}x_{1}+\beta_{2}x_{2}+\beta_{3}x_{3}+\ldots +\beta_{p}x_{p})+u,\\ \epsilon^{\left(0\right)} & = & y-\mu^{\left(0\right)}. \end{array}$$ (8)

Under the alternative hypothesis with ***θ*** vector of change points, SCP-SIM takes the following form:

$$\begin{array}{lll} y|\mu^{\left(1\right)} & \sim & \text{Pois}(\mu^{\left(1\right)}|\beta ,\theta ,u),\\ \mu^{\left(1\right)} & = & f(\beta_{1}x_{1}+\beta_{11}{\left[x_{1}-Z\theta^{1}\right]}_{+}+\ldots +\beta_{1L}{\left[x_{1}-Z\theta^{L}\right]}_{+}+\beta_{2}x_{2}+\beta_{3}x_{3}+\ldots \\ \; & \; & +\beta_{p}x_{p})+u,\\ \epsilon^{\left(1\right)} & = & y-\mu^{\left(1\right)}. \end{array}$$ (9)

The test statistic is based on the ratio of the residuals of the original data,

$$\begin{array}{c} T_{y_{\left(0\right)}}=\frac{{\left[{\hat{\epsilon }}_{y_{\left(0\right)}}^{\left(0\right)}\right]}^{\prime }\left[{\hat{\epsilon }}_{y_{\left(0\right)}}^{\left(0\right)}\right]}{{\left[{\hat{\epsilon }}_{y_{\left(0\right)}}^{\left(1\right)}\right]}^{\prime }\left[{\hat{\epsilon }}_{y_{\left(0\right)}}^{\left(1\right)}\right]}, \end{array}$$ (10)

where ${\hat{\epsilon }}_{y_{\left(0\right)}}^{\left(0\right)}$ and ${\hat{\epsilon }}_{y_{\left(0\right)}}^{\left(1\right)}$ denote the residuals under the null and alternative hypotheses of the actual data **y**_0_. Permutation-based *p*-value can be calculated and the candidate spatially dependent change points are declared significant if *p*-value <*α*, where *α* is the significant level.

When multiple change points are considered, *L* > 1, *H*_0_: *L*_0_ = 0 versus *H*_1_: *L*_1_ = *L*, where *L* is the possible number of change points, is tested. If *H*_0_ is rejected, we then test *H*_0_: *L*_0_ = 1 versus *H*_1_: *L*_1_ = *L*; otherwise, we test *H*_0_: *L*_0_ = 0 versus *H*_1_: *L*_1_ = *L* − 1 until we reach testing *H*_0_: *L*_0_ = *l* versus *H*_1_: *L*_1_ = *l* + 1. For the last two hypotheses, if *H*_0_, is rejected, then the number of significant change points declared is *l* + 1, otherwise, it is *l*.

## Simulation studies

Three simulation studies are conducted to evaluate the performance of our approach in detecting and testing change points. We assume that the number of change points is unknown and fixed. We first determine the potential maximum number of change points, *K*_*max*_, and then conduct the permutation test to identify the number of significant change points. We consider the following three cases: (1) when there are no change points, (2) when there is only one change point, and (3) when there are two change points. Using the likelihood ratio test proposed by [11], we determined *K*_*max*_. We can treat the first case with zero significant changes out of *K*_*max*_ as a type I estimated error. The second case is considered to have occurred when one change point is significant out of *K*_*max*_ and the other change points are not significant. The third case is considered to have occurred when the two change points are significant and the other change points are not significant.

### Simulation Study 1: No change points—Type I error

Type I error is studied by simulating 100 data sets from the CP-SISM with no change points (the model under the null hypothesis) that takes the form:

$$\begin{array}{ccc} y_{is} & ∼ & \text{Pois}\left(\mu_{is}|\beta ,u_{s}\right),\\ \mu_{is} & = & f\left(\beta_{1}x_{1is}+\beta_{2}x_{2is}+\beta_{3}x_{3is}\right)+u_{s}, \end{array}$$ (11)

i=1,2,…,nands=1,2,…,r,

with six locations (*r* = 6) and 100 observations at each location (*n* = 100). We set the true parameters as ***β*** = (*β*_1_, *β*_2_, *β*_3_) = (−0.5, 1, 1), *σ*_*u*_ = 1, so the mean function is equal to ***μ***_*s*_ = *f*(*X* ***β***) + *u*_*s*_ = (−0.5*x*_1_ + *x*_2_ + *x*_3_)^2^ + *u*_*s*_. In this setting, there is no change point. The permutation test is used to detect any significant change point and it is found that the null hypothesis is rejected 6 times out of the 100. This means that the Type I error of the test is maintained approximately at 5%.

### Simulation Study 2: A single change point at each city

In this section, two cases are considered: (1) there is one common change point for all cities, and (2) there are different change points for cities.

#### One common change point for all cities

One hundred data sets are simulated from the proposed model (CP-SISM), in which

$$\begin{array}{ccc} y_{is} & ∼ & \text{Pois}\left(\mu_{is}|\beta ,\theta ,u_{s}\right),\\ \mu_{is} & = & f\left(\beta_{1}x_{1is}+\beta_{11}{\left[x_{1is}-\theta \right]}_{+}+\beta_{2}x_{2is}+\beta_{3}x_{3is}\right)+u_{s}, \end{array}$$ (12)

i=1,2,…,nands=1,2,…,r,

with six locations (*r* = 6) and 100 observations at each location (*n* = 100). Three explanatory variables (*x*_1_, *x*_2_, and *x*_3_) are generated from Uniform(*π*, 2*π*). We set true parameters ***β*** = (*β*_1_, *β*_2_, *β*_3_, *β*_11_) = (−0.5, 1, 1, 1), (*θ*, *σ*_*u*_) = (4.7, 1), and the mean function ***μ***_*s*_ = *f*(*X*(***θ***)***β***) + *u*_*s*_ = (−0.5*x*_1_ + [*x*_1_ − 4.7]_+_ + *x*_2_ + *x*_3_)^2^ + *u*_*s*_. In this setting, there is a common change point at *θ*_*s*_ = *θ* = 4.7 for all *s* (*s* = 1, 2, …, *r*). Here, *β*_2_ is set to 1 to fix the identifiability problem. Based on the mean squared error (MSE), and mean, median and inter-quartile range (IQR) of the estimates, the estimation would be evaluated. In addition, the proportion of the significant detected change points is calculated.

We set the dependence range *ρ*_*u*_ = 2, and the variance of the spatial effects *σ*_*u*_ = 1. The domain of [0, 3] × [0, 3] is used in this simulation study because the range of the distance between spatial locations of latitude and longitude in the motivating data set is found to be about 2. $y_{s}|\mu_{s}$ is generated from Poisson distribution with mean ***μ***_*s*_|*u*_*s*_. The reason for not using a large value of *σ*_*u*_ in the simulation is to ensure we do not obtain a negative mean value, where the response variable has the Poisson distribution.

Table 1 shows that the mean estimates of all the parameters are close to the true values for all the parameters and change points.

**Table 1:** Results for 500 simulated data sets from CP-SISM with one common change point; the mean, median, MSE, 95% confidence interval, and empirical coverage probability of the model parameters and change point.

	True	Mean	Median	MSE	95% CI	Coverage probability
*β*_1_	-0.5	-0.5174	-0.4984	0.024	(-0.547, -0.488)	94.27%
*β*_3_	1	1.0056	1.0026	0.003	(0.9949, 1.0103)	94.07%
*β*_11_	1	1.0300	1.0172	0.029	(0.9925, 1.0419)	91.02%
*θ*	4.7	4.693	4.7000	0.054	(4.6731, 4.7137)	95.51%
*σ*_*u*_	1	0.9964	0.9620	0.159	(0.9614, 1.0314)	94.53%

To obtain the empirical coverage probability for the model parameters and the change point, 500 data sets are simulated based on the setting that is described above, and the model parameters and the change point are estimated for each simulated data set. Then, 10,000 random samples of size 30, with replacement, are selected from each parameter estimate (the model parameters and change point) and a 95% confidence interval is calculated for each parameter, for each sample. The coverage probability of each parameter is estimated by calculating the proportion of the confidence intervals that contain the true parameters. The results are reported in Table 1, which shows the confidence intervals achieve the near nominal coverage probability.

#### Different change points for cities

**Fig 3:**
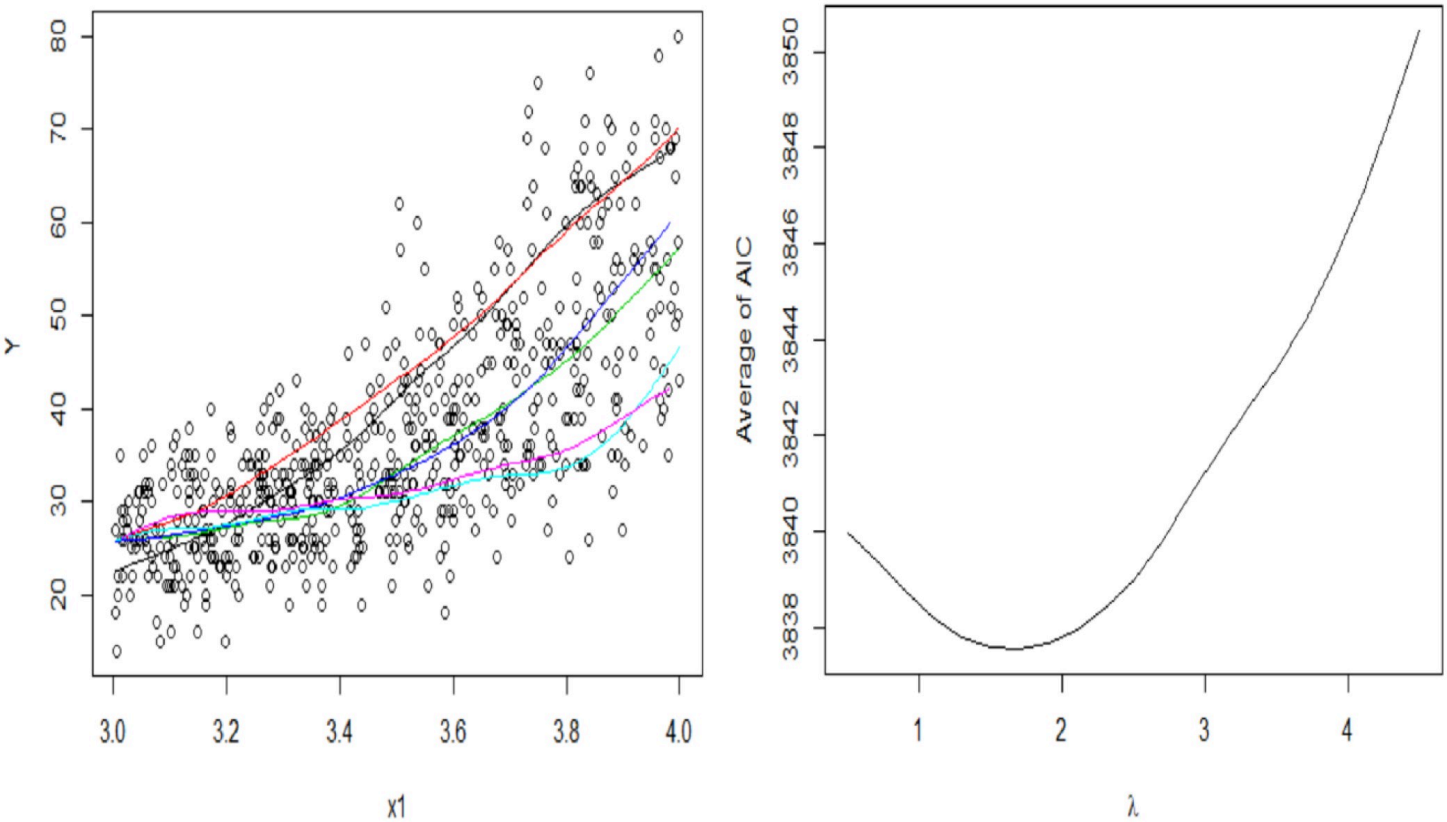
Simulated data set of six locations, every two locations has equal change points (a), and average AIC versus the penalty value, λ (b).

Similar to the simulation in Study 1, 100 data sets are simulated from the following model:

$$\begin{array}{ccc} y_{is} & ∼ & \text{Pois}\left(\mu_{is}|\beta ,\theta_{s},u_{s}\right),\\ \mu_{is} & = & f\left(\beta_{1}x_{1is}+\beta_{11}{\left[x_{1is}-\theta_{s}\right]}_{+}+\beta_{2}x_{2is}+\beta_{3}x_{3is}\right)+u_{s}, \end{array}$$ (13)

i=1,2,…,nands=1,2,…,r,

with six locations (*r* = 6), 100 observations at each location (*n* = 100). Three explanatory variables (*x*_1_, *x*_2_, *x*_3_) are generated from Uniform(3, 4). We set true parameters ***β*** = (*β*_1_, *β*_2_, *β*_3_, *β*_11_) = (1, 0.3, 0.3, 3), and the mean function *μ*_*s*_ = *f*(*X*(***θ***)***β***) + *u*_*s*_ = (*x*_1_ + 3[*x*_1_ − *θ*_*s*_]_+_ + 0.3*x*_2_ + 0.3*x*_3_)^2^ + *u*_*s*_. The data are generated such that every two locations share the same change point, three different spatial change points in total. The first two locations have a change point at 3.2 (*θ*_1_ = *θ*_2_ = 3.2), the second two locations have change points at 3.5 (*θ*_3_ = *θ*_4_ = 3.5), and the last two locations each have a change point at 3.8 (*θ*_5_ = *θ*_6_ = 3.8). The variance of the spatial effects is 1, *σ*_*u*_ = 1. The [0, 3] × [0, 3] domain is used in this simulation study. Three cases are considered for the dependence range (*ρ*_*u*_ = 0.5, 1, and 2). Here, *ρ*_*u*_ = 0.5 means there is not much dependence, and *ρ*_*u*_ = 2 means a high dependence range. **y** *_s_*|***μ***_*s*_ is generated from the Poisson distribution with mean ***μ***_*s*_|*u*_*s*_. Fig 3(a) shows a random simulated data set based on this setting. Under this setting, it is found that the spatial variance estimate is over-estimated, so a penalty value is used, λ, in the M-step of the proposed estimation algorithm.

Fig 3(b) shows the average of AIC at each value of λ. It reveals that the optimal value is about 1.9. One hundred data sets are generated from this setting, and using the optimal value of λ, the MSE, mean, median, and IQR of the estimates are calculated to evaluate the estimating approach. The permutation-based test is used to test the significance of the detected change points and the proportion of the significant detected change points is calculated as well. Table 2 shows the results of the 100 simulated data sets. It shows that the performance of the proposed model in detecting change points works well. The model parameter estimates are close to the true values and have quite small standard error and MSE. The model parameter estimates and detection of change points under different values of dependence range, *ρ*_*u*_, are comparable. The proportions of significant detected change points for the different values of the dependent range are 98% for *ρ*_*u*_ = 0.5, 97% for *ρ*_*u*_ = 1, and 97% for *ρ*_*u*_ = 2.

**Table 2:** Results of 100 simulated data sets: MSE, mean, median, and standard error of the model parameters and change points estimates for different values at different dependence range, *ρ*_*u*_ = 0.5, 1, 2.

		True	Mean ± SE	MSE	Median	IQR
*ρ* = 0.5	*β*_2_	0.3	0.31 ± 0.021	0.033	0.27	0.08
	*β*_3_	0.3	0.30 ± 0.043	0.042	0.30	0.10
	*β*_11_	3	3.09 ± 0.067	0.130	2.75	0.15
	*θ*_1_	3.2	3.21 ± 0.001	0.002	3.20	0.05
	*θ*_2_	3.5	3.51 ± 0.007	0.004	3.55	0.10
	*θ*_3_	3.8	3.78 ± 0.005	0.007	3.80	0.05
	*σ*_*u*_	1	1.03 ± 0.051	0.122	0.93	0.15
*ρ* = 1	*β*_2_	0.3	0.29 ± 0.020	0.032	0.26	0.09
	*β*_3_	0.3	0.30 ± 0.046	0.042	0.27	0.08
	*β*_11_	3	2.89 ± 0.065	0.130	2.67	0.13
	*θ*_1_	3.2	3.23 ± 0.001	0.002	3.20	0.05
	*θ*_2_	3.5	3.49 ± 0.002	0.004	3.45	0.10
	*θ*_3_	3.8	3.73 ± 0.002	0.007	3.75	0.05
	*σ*_*u*_	1	0.97 ± 0.041	0.124	0.92	0.15
*ρ* = 2	*β*_2_	0.3	0.26 ± 0.027	0.033	0.24	0.10
	*β*_3_	0.3	0.25 ± 0.095	0.046	0.24	0.11
	*β*_11_	3	2.66 ± 0.105	0.134	2.33	0.14
	*θ*_1_	3.2	3.16 ± 0.015	0.005	3.15	0.10
	*θ*_2_	3.5	3.45 ± 0.012	0.006	3.40	0.10
	*θ*_3_	3.8	3.74 ± 0.008	0.010	3.70	0.05
	*σ*_*u*_	1	0.98 ± 0.128	0.138	0.94	0.19

### Simulation Study 3: Two change points

One hundred and fifty data sets are simulated from the proposed model (CP-SISM),

$$\begin{array}{ccc} y_{is} & ∼ & \text{Pois}\left(\mu_{is}|\beta ,\theta_{s},u_{s}\right),\\ \mu_{is} & = & f\left(\beta_{1}x_{1is}+\beta_{11}{\left[x_{1is}-\theta_{1}\right]}_{+}+\beta_{12}{\left[x_{1is}-\theta_{2}\right]}_{+}+\beta_{2}x_{2is}\right)+u_{s}, \end{array}$$

i=1,2,…,nands=1,2,…,r,

with six locations (*r* = 6) and 100 observations at each location (*n* = 100). Two explanatory variables (*x*_1_ and *x*_2_) are generated from Uniform(*π*, 3*π*). We set true parameters ***β*** = (*β*_1_, *β*_2_, *β*_11_, *β*_12_) = (1, 1, −2, 1.5), (*θ*_1_, *θ*_2_, *σ*_*u*_) = (4.5, 7.5, 1), and the mean function ***μ***_*s*_ = *f*(*X*(***θ***)***β***) + *u*_*s*_ = (*x*_1_ + [*x*_1_ − 4.5]_+_ + [*x*_1_ − 7.5]_+_ + *x*_2_)^2^ + *u*_*s*_. In this setting, there are two change points at *θ*_1_ = 4.5 and *θ*_2_ = 7.5 for all locations. Here, *β*_2_ is set to 1 to fix the identifiability problem. Based on the mean, median, and MSE of the estimates and 95% confidence interval of the model parameters, the estimation would be evaluated. Table 3 shows that in the estimation based on the criteria that are used, the model parameters are well estimated.

**Table 3:** Results for 150 simulated data sets from CP-SISM with two change points; the mean, median, MSE, and 95% confidence interval of the model parameters and change point.

	True	Mean	Median	MSE	95% CI
*β*_1_	1	0.991	0.990	0.092	(0.943, 1.038)
*β*_11_	-2	-2.002	-1.986	0.077	(-2.047, -1.959)
*β*_12_	1.5	1.507	1.497	0.077	(1.480, 1.536)
*θ*_1_	4.5	4.527	4.500	0.033	(4.498, 4.557)
*θ*_2_	7.5	7.471	7.500	0.034	(7.439, 7.504)
*σ*_*u*_	1	1.112	1.095	0.220	(1.045, 1.178)

## Real data application

In this section, our approach is applied to our motivating data. Non-accidental mortality (ICD-10 codes A00-R99) data are obtained from Statistics Korea and historical weather data, such as daily average temperature, pressure, and humidity, are obtained from the Korea Meteorological Administration. Non-accidental mortality (excluding deaths related to accidents) is chosen because it has been widely used in previous studies. The non-accident mortality and weather variables were recorded daily from January 2000 to December 2007 for six major cities in South Korea: Busan, Daegu, Daejeon, Gwangju, Incheon, and Seoul. The total number of records is 2,922 days with 668,583 deaths. The weekly data are also created from this daily data, which resulted in 417 observations at each city. The latitude and longitude in this motivating data set are further explained in S1 Table of the supporting information.

In previous studies, a change point was estimated for each city separately and a common change point was considered [16, 18, 20, 36]. In addition, testing the change point was not conducted.

Our goals are simultaneously (1) estimate the relationship between the weekly non-accident mortality (**y**) and mean temperature (**x**_1_), adjusting for other covariates such as mean humidity (**x**_2_), mean pressure (**x**_3_), and month as a factor (**x**_4_); (2) to detect the possible spatially dependent change points in temperature of each city; and (3) to test whether the detected spatial change points are significant by using the proposed permutation-based test. In our weekly motivating data, we have four explanatory variables (*p* = 4) and 417 observations for each city (*n* = 417).

### Detecting and testing a common change point

The proposed model, with a common change point of all cities *θ*_*s*_ = *θ*, has the form

$$\begin{array}{ccc} y_{s} & ∼ & \text{Pois}\left(\mu_{s}|\beta ,\theta ,u_{s}\right),\\ \mu_{s} & = & f\left(\beta_{1}x_{1}+\beta_{11}{\left[x_{1}-\theta \right]}_{+}+\beta_{2}x_{2}+\beta_{3}x_{3}+\beta_{4}x_{4}\right)+u_{s}, \end{array}$$ (14)

and with no common change point, it takes the form

$$\begin{array}{ccc} y_{s} & ∼ & \text{Pois}\left(\mu_{s}|\beta ,\theta ,u_{s}\right),\\ \mu_{s} & = & f\left(\beta_{1}x_{1}+\beta_{2}x_{2}+\beta_{3}x_{3}+\beta_{4}x_{4}\right)+u_{s},\;s=1,2,\ldots ,r. \end{array}$$ (15)

One common change point is detected. We then test whether this detected change point is significant. The results are compared to the case of aggregating the data of all cities by ignoring spatial effects.

Table 4 shows that the proposed model, CP-SISM, fits the data better than the aggregated model does, in which the *R*^2^ (=0.69) of CP-SISM is much higher than *R*^2^ (=0.22) of the aggregated data model is. However, the same change point value (*θ* = 22^*o*^*C*) is detected and found to be significant, but the *p*-value of the proposed model is smaller. The standard error and confidence intervals of the parameters and change point are calculated using a permutation approach as follows:

**Table 4:** Parameter estimates, standard errors, change points detected, p-values, and *R*^2^ of CP-SISM and the aggregated data model, assuming there is one common change point.

	Aggregated Model	CP-SISM
${\hat{\beta }}_{1}$	-1.769±0.035	-1.334±0.034
${\hat{\beta }}_{11}$	2.439±0.036	1.782±0.033
${\hat{\beta }}_{2}$	.0963±0.001	-0.145±0.0003
${\hat{\beta }}_{4}$	-0.4852±0.018	0.319±0.0048
${\hat{\sigma }}_{u}$	—	29.99
*θ*±*SE*(*θ*)	22 ± 0.124	22 ± 0.111
p-value	0.019	0.009
*R* ^2^	0.22	0.69

Step 1. A sample of observations from each city (with replacement) is randomly selected.
Step 2. The model parameters and the change point are estimated.
  1. Step 1—Step 2 are repeated 500 times and the standard error and confidence interval are calculated for each parameter and change point.

**Fig 4:**
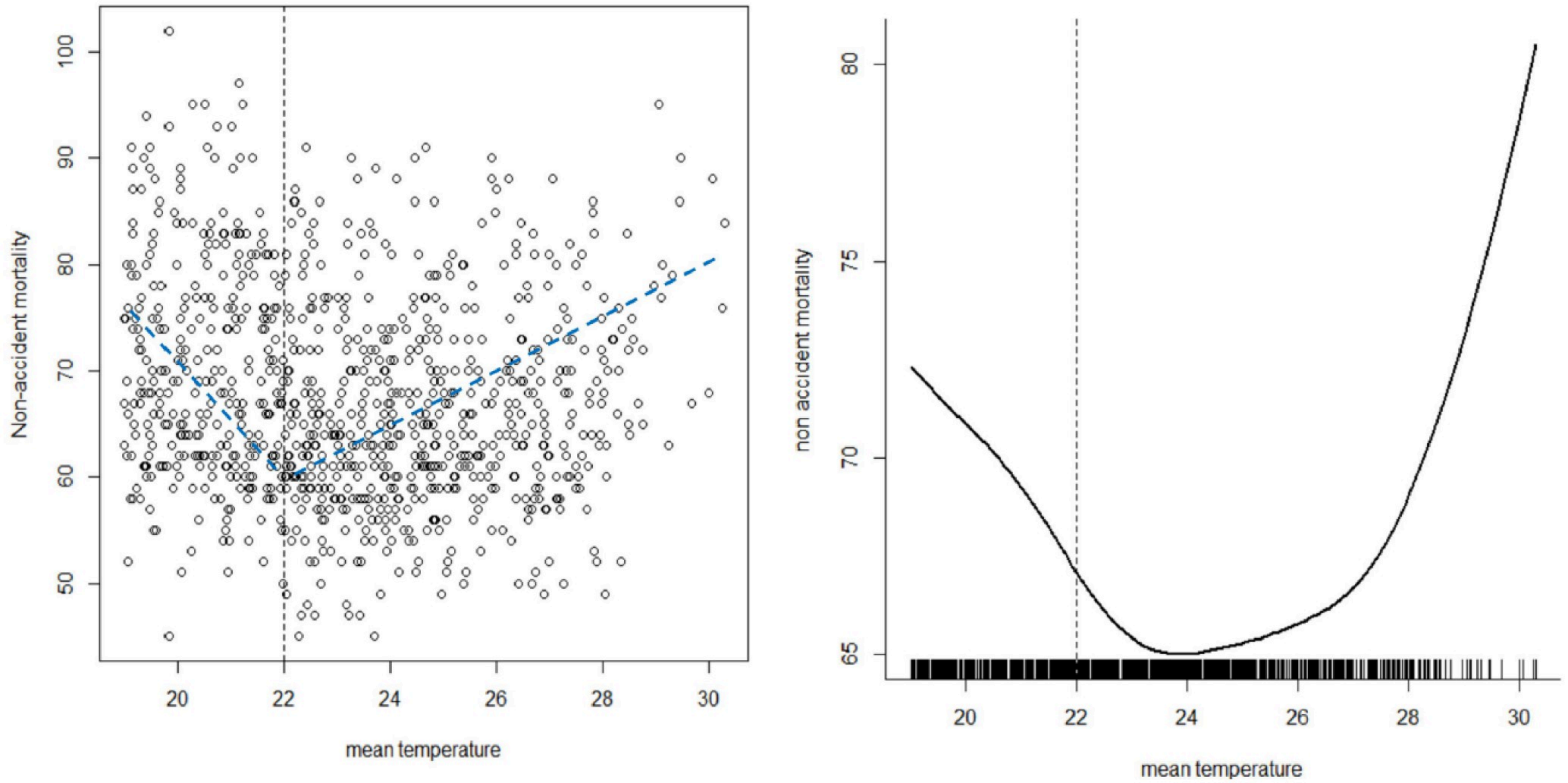
Scatter plot along with the detected common change point (a), Spline smoothed mortality-temperature function of all the six cities (b).

**Fig 5:**
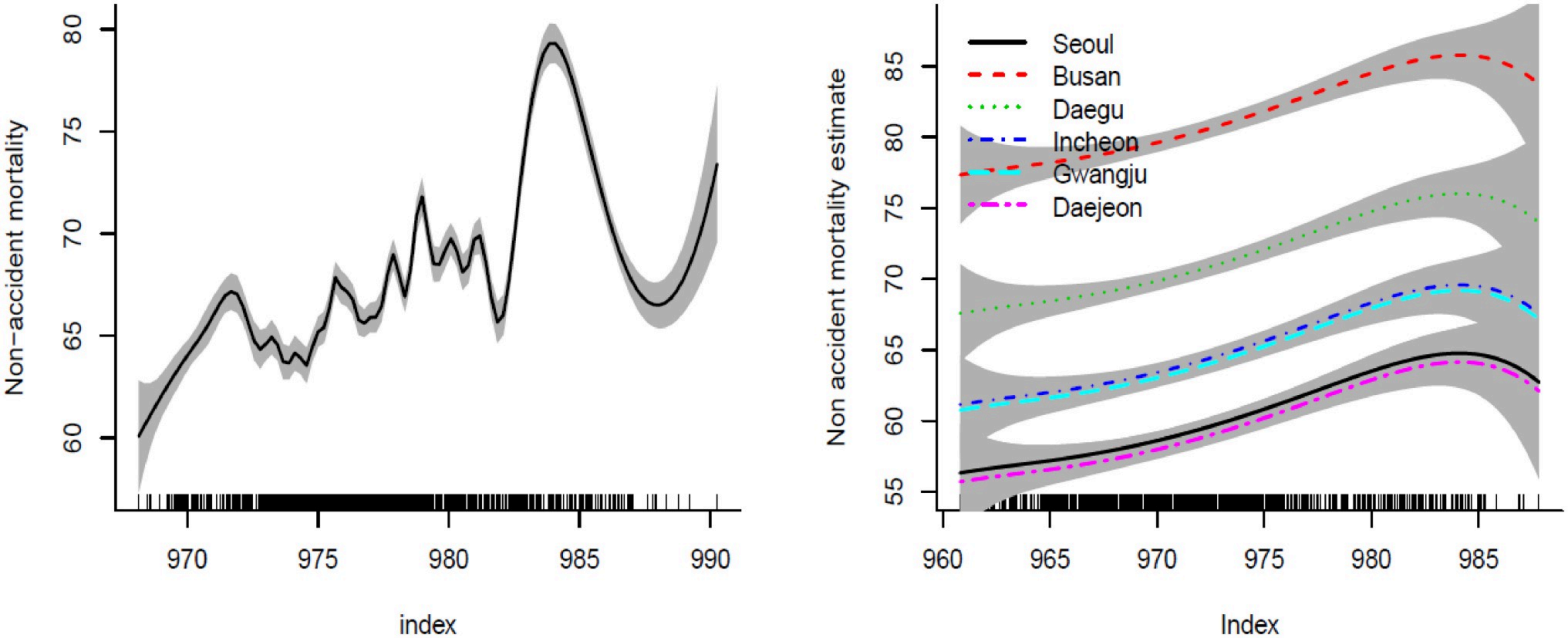
Smoothed mortality function of the aggregated data of the six cities (a) and the smoothed mortality functions from the proposed model (b).

It is found that the change point estimate of the proposed model has a smaller standard error compared to that of the aggregated model. Fig 4 shows that detecting the change point by smoothing the unknown function does not give an accurate value. The change point by smoothing the unknown function is about 24^*o*^*C*. However, based on the permutation test, the change point of the proposed model is about 22^*o*^*C*. Fig 4(a) shows the smoothed function for aggregated data and Fig 4(b) shows the smoothed functions for the six cities from the proposed model. The smoothed function of the aggregated data is wigglier compared to the smoothed functions of the proposed model. Fig 5(b) shows that the highest mortality is for Busan and the lowest mortality function is for Gwangju and Seoul.

Regarding the single index coefficient estimates and their standard errors, Table 4 shows that the standard errors estimated by the permutation method for the proposed model are smaller than those of the aggregated model. We also noticed that some of the coefficients are different in the sign. For both models, the coefficient of the mean pressure is set to 1 to fix the identifiability problem.

### Detecting and testing various change points

The proposed model has the following form:

$$\begin{array}{ccc} y_{s} & ∼ & \text{Pois}\left(\mu_{s}|\beta ,\theta_{s},u_{s}\right),\\ \mu_{s} & = & f\left(\beta_{1}x_{1}+\beta_{11}{\left[x_{1}-\theta_{s}\right]}_{+}+\beta_{2}x_{2}+\beta_{3}x_{3}+\beta_{4}x_{4}\right)+u_{s},\;s=1,2,\ldots ,r. \end{array}$$ (16)

Simultaneously, the model is estimated, the spatially dependent possible change points are detected, and the detected change points are tested to determine whether they are significant. The results are compared to the case of detecting a change point in each city separately and then tested to determine whether it is significant.

Table 5 shows the change points that are detected in case of no spatial effects (each city is analyzed separately) and the proposed model, CP-SISM, along with the standard errors and *p*-values. It is found that the change points that are detected and tested simultaneously are comparable to the no spatial effects case. However, the parameter estimates of the CP-SISM have smaller standard errors.

**Table 5:** Detected change points in the two cases: No spatial effects considered (change point is detected and tested for each city separately), and spatial effect considered in our proposed model, CP-SISM (change points are detected and tested simultaneously).

	No spatial		CP-SISM
	${\hat{\theta }}_{s}\pm se\left({\hat{\theta }}_{s}\right)$	p-value	${\hat{\theta }}_{s}\pm se\left({\hat{\theta }}_{s}\right)$
Busan	23.2±0.131	0.371	23.2±0.112
Incheon	22.8±0.132	0.148	22.8±0.122
Seoul	22.6±0.136	0.019	22.4±0.124
Daegu	22.6±0.133	0.059	22.6±0.121
Daejeon	22.6±0.142	0.029	22.6±0.109
Gwangju	22.4±0.131	0.049	22.4±0.113
			*R*^2^ = 0.73 and p-value = 0.000

Under the CP-SISM, the smallest change point value is for Seoul and Gwangju (22.4), and the highest change point value is for Busan (23.2). In addition, for the no spatial effects case, one can see that three of the cities have insignificant change points (Incheon, Busan, and Daegu). For the CP-SISM, *R*^2^ is improved (*R*^2^ = 0.73) compared to the one common change point case (*R*^2^ = 0.69). The improvement is not significant because the detected change points are close and close to the common change point value except Busan city change point which has a higher change point compared to the other cities. As a result, the difference between the two models’ *R*^2^ values is not big.

**Fig 6:**
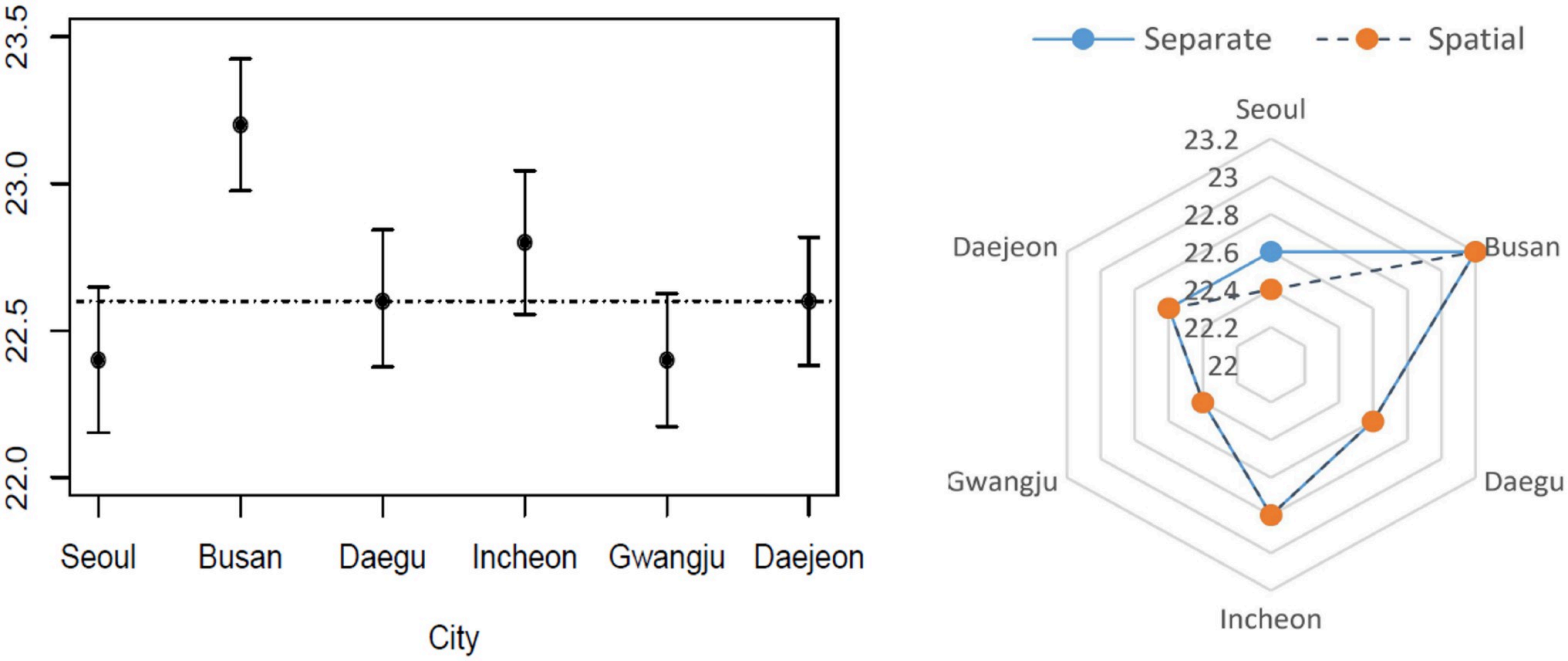
The 95% confidence interval for each change point (a) and a radar plot of the significant detected change points using the proposed model (CP-SISM) and no spatial random effects considered case (b).

Fig 6(a) compares the change points detected in each city under the CP-SISM and under the case of detecting a change point in each city separately. To check whether these change points of the CP-SISM are different, the 95% confidence interval for each detected change point is calculated using the permuted standard error and shown in Fig 6(b). It reveals the confidence intervals of the detected change points overlap, except for Busan city. This explains why there is not much difference between the two cases: one common change point and the spatially dependent change points case. These two cases are also compared in terms of the model parameter estimates and the results are summarized in Table 6. It shows they have comparable parameter estimates and standard errors, as well as comparable estimate values of spatial effects. It shows that the smallest spatial random effects are of Seoul and Daejeon and the highest is of Busan, which is much higher compared to the other cities. Busan has the highest change point value and the highest mortality.

**Table 6:** Parameter estimates and standard errors, spatial effect estimates of the proposed model (CP-SISM) in case one common spatial change point is assumed, *θ*, and in case different spatially-dependent change points are assumed, *θ* = (*θ*_1_, …, *θ*_6_).

		CP-SISM	CP-SISM
		A common change point	Spatially-dependent change points
Parameter estimates	${\hat{\beta }}_{1}$	-1.334± 0.035	-1.436± 0.032
	${\hat{\beta }}_{11}$	1.782± 0.036	2.104± 0.031
	${\hat{\beta }}_{2}$	-0.145±0.001	-0.145± 0.001
	${\hat{\beta }}_{4}$	0.319± 0.018	0.2921±0.011
Spatial effects	Busan	13.28	13.99
	Incheon	-2.91	-2.18
	Seoul	-7.73	-7.11
	Daegu	3.53	4.30
	Daejeon	-8.35	-7.56
	Gwangju	-3.29	-2.73

## Discussion and conclusion

A semiparametric regression model (CP-SISM) is introduced to simultaneously estimate the nonlinear temperature-mortality relationship, detect spatially dependent change points, and test to determine whether they are significant based on a permutation-based test, and a unified method is proposed. Simulation studies are conducted for two cases: change points are spatially independent and change points are spatially dependent. Simulation studies showed that our approach works well in estimating, detecting, and testing spatial change points simultaneously.

The advantages of our proposed approach are demonstrated using epidemiology data on mortality and temperature, as well as other weather variables that were collected daily from six major cities in South Korea. It is found that cities have close change points, except Busan city, which has a higher change point value and higher mortality. The proposed model, CP-SISM, with one common change point for all cities, is compared to the aggregated data model that is commonly used in previous studies, and the proposed model was found to be much better in terms of fitting the data (higher *R*^2^) and detecting the significant spatial change point (smaller *p*-value and standard error). The proposed model, CP-SISM, with possible spatially dependent change points, is compared to the case of each city’s data separately analyzed to detect its change points, which is considered in many previous studies. It is found that the change-point values are comparable, but three cities have insignificant change points in the case of separately analyzed city data (previous studies have not tested the change points detected) and the change-point estimates of the CP-SISM have smaller standard errors and smaller *p*-values.

The proposed model with one common change point is compared to the spatially dependent change points case. Both models showed that Busan City has the highest mortality, and Seoul and Daejeon have the lowest mortality. The CP-SISM with spatially-dependent change points has a higher *R*^2^ value and detected that one of the cities (i.e., Busan) has a higher change point compared to the other cities.

The proposed model offers several opportunities for extension and enhancements to improve the estimation method. The proposed model assumes the mean mortality function over the cities has the same shape; however, this assumption can be relaxed and can use different functions for different cities.

In the proposed model, it is assumed that the mean mortality functions, *f*(⋅), over cities, have the same form and then detect and test the change points. It is possible to consider change point detection for the non-parametric part *f*(⋅). This approach would involve identifying shifts in the functional form or underlying structure of *f*(⋅) over locations. Implementing change point detection in a non-parametric context, however, may require different techniques, or other non-parametric hypothesis tests, to effectively capture and detect changes in *f*(⋅). The model assumes that the slopes before and after the change point are the same for all cities, but different slopes can be used. In the model estimation, a grid search is used to obtain the change points, however, better methods can be used such as assuming the change points are random variables following some distribution with some mean and variance, such as a normal distribution. This will reduce the estimation time, especially if the Bayesian approach is used. The proposed approach is applied to 6 cities in South Korea, but it can be applied to cities from different countries. In some countries, the spatial effects may be integrated into the mean function as follows:

$$\begin{array}{ccc} \mu |u,\beta ,\theta & = & f(X(\theta )\beta +Zu). \end{array}$$

In this case, there will be no identifiability problem for the single index function, and for some countries, the spatial random effects may not be additive to the nonparametric function. Mortality was found to depend on pollutant and weather variables as an index (a linear combination of these variables). In this context, a variable selection method can identify significant index variables affecting mortality. To address the identifiability issue and facilitate variable selection, the constraint ||*β*|| = 1 can be applied instead of fixing the first parameter of *β* to 1. The proposed model can be extended to accommodate generalized linear models beyond the Poisson framework. For instance, when the response variable is binary, methods designed for estimating single-index functions can be applied using Bernoulli distribution. Once the single-index model is estimated using such an approach, the subsequent steps in the proposed methodology become straightforward.

Environmental epidemiology often provides high-dimensional variables so we need to detect many change points. In this case, we can build a high-dimensional nonparametric model using deep neural network tools and visualize these high-dimensional change points using computer vision. These connections among machine learning architecture [37], computer vision [38, 39], and statistical models will provide more flexible analytical tools for complex data.

## Supporting information

S1 Table Characteristics of the 6 major cities in Korea: Seoul, Busan, Daegu, Incheon, Gwangju, and Daejeon. (PDF)
