# Supplementary material for: Semiparametric change points detection using single index spatial random effects model in environmental epidemiology study
Source: PLoS One. 2024 Dec 12;19(12):e0315413. doi: 10.1371/journal.pone.0315413 (PMC11637292; doi:10.1371/journal.pone.0315413)
Supplement: S1 Table — (PDF) [file pone.0315413.s001.pdf]

Supporting information for "Semiparametric Change Points Detection Using Single Index Spatial Random Effects Model in Environmental Epidemiology Study"

Hamdy F. F. Mahmoud[1,2*] and Inyoung Kim[1]

[1] Department of Statistics, Virginia Polytechnic Institute and State University (Virginia Tech), VA 24061.

[2] Department of Statistics, Mathematics, and Insurance, Assiut University, Assiut 71515, Egypt.

*To whom correspondence should be addressed:

Hamdy F.F. Mahmoud, Ph.D

Department of Statistics, Virginia Tech., Blacksburg, VA 24061, USA.

E-Mail: ehamdy@vt.edu

Table 1: Characteristics of the 6 major cities in Korea: Seoul, Busan, Daegu, Incheon, Gwangju, and Daejeon

	Latitude	Longitude	Population in 2007	Mean temperature	Mean daily mortality
Seoul	$37^o\ 34^{'}$	$126^o\ 58^{'}$	10,192,710	12.87	93.16
Busan	$35^o\ 06^{'}$	$129^o\ 02^{'}$	3,587,439	14.78	45.1
Daegu	$35^o\ 53^{'}$	$128^o\ 37^{'}$	2,493,261	14.5	27.39
Incheon	$37^o\ 28^{'}$	$126^o\ 38^{'}$	2,664,576	12.73	25.97
Gwangju	$35^o\ 10^{'}$	$126^o\ 54^{'}$	1,413,444	13.98	14.00
Daejeon	$36^o\ 22^{'}$	$127^o\ 22^{'}$	1,475,659	13.13	13.67
